# An eye on semantics: a study on the influence of concreteness and predictability on early fixation durations

**DOI:** 10.1080/23273798.2023.2274558

**Published:** 2023-11-09

**Authors:** Federica Magnabosco, Olaf Hauk

**Affiliations:** MRC Cognition and Brain Sciences Unit, University of Cambridge, Cambridge, UK

**Keywords:** semantic cognition, reading, context, eye movements, predictability, concreteness

## Abstract

We used eye-tracking during natural reading to study how semantic control and representation mechanisms interact for the successful comprehension of sentences, by manipulating sentence context and single-word meaning. Specifically, we examined whether a word’s semantic characteristic (concreteness) affects first fixation and gaze durations (FFDs and GDs) and whether it interacts with the predictability of a word. We used a linear mixed effects model including several possible psycholinguistic covariates. We found a small but reliable main effect of concreteness and replicated a predictability effect on FFDs, but we found no interaction between the two. The results parallel previous findings of additive effects of predictability (context) and frequency (lexical level) in fixation times. Our findings suggest that the semantics of a word and the context created by the preceding words additively influence early stages of word processing in natural sentence reading.

## Introduction

Semantic knowledge is central to our everyday life: our interaction with the world relies on previous knowledge about objects, people, facts, and abstract concepts. *Semantic cognition* refers to the representation of acquired knowledge about the world and its controlled and task-oriented use (Patterson et al., 2007). From a cognitive neuroscience perspective, the Controlled Semantic Cognition (CSC) framework has recently been proposed in order to explain how the knowledge of concepts is represented and flexibly used (Lambon Ralph et al., 2017). The CSC argues that the successful comprehension of meaningful stimuli relies on two interactive systems: semantic control and semantic representation. The semantic representation system is responsible for the multimodal representation of concepts. It relies on the interaction between a central transmodal hub, located in the anterior temporal lobe (ATL), and a distributed network of modality-specific regions (Patterson et al., 2007; Patterson & Lambon Ralph, 2016). On the other hand, a semantic control system comprising prefrontal and posterior-temporal areas enables the task- and context-appropriate activation of semantic information, manipulating the activation within the semantic representation system (Jackson, 2021). As the interaction between these two systems makes flexible context-dependent semantic cognition possible, studying the dynamics of this interaction in both brain and behaviour is an important step for the development of theories of semantic cognition. Most frequently, the study of concept representation has relied on the observation of behavioural and neural measures following the presentation of items in isolation. Here, we propose sentence reading as a step towards studying natural language processing using a naturalistic yet well-controlled experimental paradigm.

In our study, we aim at investigating the relationship between semantic control and representation by focusing on the effects of context and single-word semantics during natural reading. Specifically, we simultaneously examined the effects of word predictability and concreteness, respectively representing sentence context and single-word semantic features. We decided to focus on predictability because it taps into both top-down as well as bottom-up semantic control: if a word is easy to predict, then it will also be easy to integrate with the preceding context due to the context-driven activation of semantically related concepts and features. On the other hand, when a word is unpredictable, this pre-activation is not present or at least reduced (Kuperberg & Jaeger, 2016). In other words, the context facilitates some concepts more than others; when a word is unpredictable or surprising, a control mechanism is necessary to retrieve and select the context-relevant semantic features of the word.

The concreteness of a word has been shown to produce reliable effects in semantic brain areas such as the ATL in neuroimaging research (Binder et al., 2005; Dhond et al., 2007; Farahibozorg et al., 2022; Hoffman et al., 2015). Behavioural evidence has shown that abstract words take longer to process than concrete ones (Bottini et al., 2022; Juhasz & Pollatsek, 2011; Richards, 1976) and are more disrupted in semantic dementia, a neurodegenerative disease that selectively affects semantic representations (Hoffman, Jones, et al., 2013).

Why abstract words are more challenging than concrete words is still controversial. The context availability theory suggests that abstract words are more difficult because of a smaller amount of semantic contextual information they can rely on (Schwanenflugel et al., 1988; Schwanenflugel & Shoben, 1983; Schwanenflugel & Stowe, 1989). Other authors have suggested that while concrete concepts are characterised by a unitary focal referent, abstract words are associated with more inhomogeneous representations and therefore are likely to have more complex representations (Pexman et al., 2007). In other words, abstract word representations are more complex because they are experienced in a wide range of physical, emotional, and mental situations, contrary to concrete objects (Barsalou & Wiemer-Hastings, 2005). A corollary is that abstract words’ representations should be more sensitive to context, as the specific meaning that is intended with a certain abstract word changes in different situations (Hoffman, 2016). Both theories predict that the effort associated with abstract words should be smaller when they occur in a predictive context: In a predictable context, the context-relevant semantic features will at least partly be preactivated and will therefore require less effortful retrieval and selection processes, which should especially benefit abstract words with more variable representations. In support of this view, the concreteness advantage in word naming and lexical decision tasks seems to disappear in predictable sentence contexts (Schwanenflugel et al., 1988; Schwanenflugel & Shoben, 1983). To our knowledge, this finding has not yet been replicated in eye movements.

### Semantic cognition during sentence comprehension

Some previous studies have focused on semantic influences on visual word recognition as measured by eye movements during natural reading (Juhasz & Pollatsek, 2011). Most of them examined sentence-level meaning and how it affects early reading times by means of predictability (see Staub, 2015, for a review), plausibility (Rayner, Warren, et al., 2004), and ambiguity (Duffy et al., 1988). In particular, the effects of predictability on early reading time measures such as first fixation duration (FFD), gaze duration (GD) and the probability of fixating a word (PrF) have been well established. Predictability is believed to act by pre-activating likely alternatives, and the level of pre-activation is modulated by the words’ cloze probabilities (Staub, 2015), without inhibiting words that are unlikely to appear (Frisson et al., 2017).

At a single-word level, lexical and linguistic factors that are known to influence fixation durations include word frequency, number of meanings, and phonological characteristics (see Kliegl et al., 2006, and Rayner, 2009, for a review of these and other factors). However, only a few studies have investigated the effects of semantic single-word variables on fixation durations. In a multiple regression study investigating lexical effects on eye movements by Juhasz and Rayner (2003), the authors included concreteness, among other variables, as a predictor. The authors showed for the first time that concreteness influenced FFD, GD and total fixation duration; however, the effect depended on which measure of frequency was included in the model. A subsequent study examined the possible effects of lexical and semantic variables on compound words (Juhasz, 2018). It appeared that sensory experience ratings but not imageability influenced early fixation durations. Finally, one study investigated the relationship between emotional, sensorimotor and linguistic characteristics during natural reading (Sheikh & Titone, 2013). The authors concluded that eye movement behaviour is modulated by both emotional and sensorimotor information, especially for low-frequency words.

While the interaction between context and single-word semantics has not been investigated yet, a number of studies have considered the joint effects of predictability and lexical word frequency on eye movements (e.g. Hand et al., 2010; Rayner, Ashby, et al., 2004). Following a similar logic as for predictability and concreteness, we might expect that the word frequency effect is diminished for highly predictable compared to unpredictable contexts. However, a common finding is that their relationship is additive (Rayner, Ashby, et al., 2004; Staub, 2011, 2015). Importantly, this is in contrast with the interactive influence these variables have on ERPs, where a predictive context eliminated the frequency effect of a larger N400 for low-frequency words (Dambacher et al., 2006; Van Petten & Kutas, 1991). Our study will provide a similar evidence base for concreteness effects in context, which can then be further investigated with neuroimaging and in particular EEG/MEG methodology.

### Aims and hypotheses

Our study has two main aims: (1) to corroborate the inconsistent previous findings of concreteness effects on eye movement data, and (2) to test whether eye movements during natural reading of naturalistic sentences are sensitive to the interaction of context and single-word meaning. We want to test whether abstract words are associated with longer reading times than concrete words, and whether a concreteness effect interacts with a word’s predictability, e.g. whether rich contextual information overrides concreteness. From a CSC perspective, finding an interaction between predictability and concreteness would provide support for early interactive involvement of both control and representation during natural reading. However, such an interaction has not been established for lexical frequency in eye movements either, while it appears to be present in evoked brain responses. Our current behavioural study will therefore be a basis for the interpretation of neuroscientific investigations into the interaction of context and semantics and provide a behavioural basis for future investigations in this area.

## Methods

### Participants

We recruited 41 participants (31 women, 9 men, 1 other/preferred not to respond; mean age 28 ± standard deviation 10 years, range 18–62). All participants reported to be native English speakers, to have normal or corrected-to-normal vision, and to have no history of neurological, developmental or language disorder. They were monetarily reimbursed for their participation in the study. One participant did not complete the study due to technical problems during the recording, so we analysed data from 40 participants. This study was approved by the Cambridge Psychology Research Ethics Committee.

### Stimuli

We created 400 sentences each containing a target word towards the end of the sentence. The total number of words in each sentence ranged between 8 and 17 (average = 12). The position of the target word in the sentence was always between the 6th and the 12th word (average position 8.5), and it was always followed by at least two more words. For more information about the stimuli, please visit this repository (https://osf.io/wyp7a/).

#### Online rating study

We measured the predictability of the target word with an online cloze probability rating study (Taylor, 1953) created and presented using the toolboxes jsPysch (de Leeuw, 2015) and JATOS (Lange et al., 2015). We split the 400 sentences in half so that the stimuli were divided into two sets of 200 sentences each. This was necessary to keep the duration of the study under one hour. Each set was presented to 50 participants recruited on Prolific (http://www.prolific.co/). The task consisted in presenting a frame of a sentence until the word preceding the target to the participants, who were asked to type the word they considered the most likely to come next. After their answer, the full sentence that was intended for the eye-tracking study was shown and participants were asked to rate how plausible were the events presented in that example. The order of presentation was fully randomised across participants. They were monetarily reimbursed for their participation in the study. During data collection, one participant was not able to conclude the study due to a server error. After a first analysis, we detected 2 participants who did not complete the task appropriately for a large part of the study (either by not providing any word, or words consistently unrelated to the context) and excluded them from the cloze calculations. Around half of the sentences turned out to be highly unpredictable (cloze value median = 0.10, mean_ _= 0.28, range_ _= [0, 1]) and sentences were generally considered plausible (plausibility value median = 5.97, mean_ _= 5.78, range_ _= [2.8, 6.77] where the Likert scale was [1, 7]).

#### Semantic predictability

**Figure 1.**
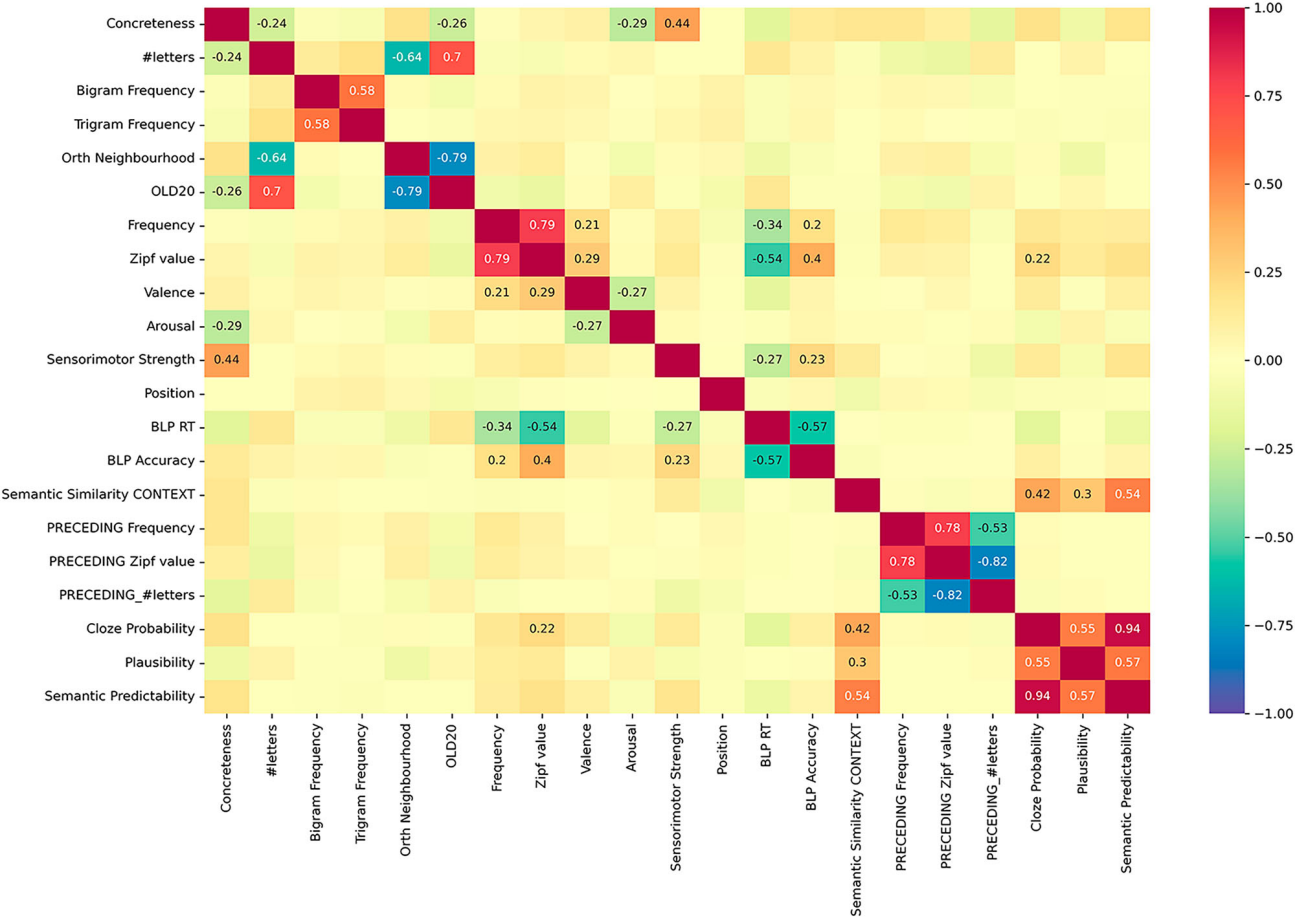
Intercorrelation matrix of relevant semantic, psycholinguistic, and context variables. All correlations greater than |.2| are reported numerically in the matrix.

We analysed the semantic similarity between the responses that were provided and the target word in more detail, for two reasons: (1) some participants provided more than one word, sometimes including adjectives before a noun, or completed the sentence; (2) some participants did not provide the exact word, but words that were semantically similar to the target word, and perfectly coherent with the sentence frame. We wanted to be able to capture that semantic predictability effects in eye movements result from a graded activation of potentially many words, rather than a discrete prediction of a specific word (Staub, 2015). Therefore, we measured the predictability of our target words as the similarity between the words provided by the participants and the target word as measured by the word2vec similarity algorithm (Mikolov, Chen, et al., 2013), pre-trained on Google News dataset (Mikolov, Sutskever, et al., 2013; Řehůřek & Sojka, 2010). Specifically, for each sentence we calculated the semantic similarity between the target word and the words produced by each participant; we selected for each participant’s response the most similar word (this allowed us for example to focus on the word of interest when it was preceded by an adjective) and then calculated the average semantic predictability across participants. For example, if the word provided by a participant was the same as the target, the semantic similarity was 1, and if many participants provided the same word, also the semantic predictability was close to 1. On the other hand, when the words produced were consistently unrelated to the target, the semantic predictability was closer to 0. In practice, cloze and semantic predictability were highly correlated (as can be seen in Figure 1) and in fact, produced similar results (see Appendix) (Table 1).

**Table 1.** Examples of the stimuli, including the associated concreteness (range 1, highly abstract, to 5, highly concrete) and predictability (range 0, highly unpredictable, to 1, highly predictable).

Sentence	Concreteness (1–5)	Predictability (0–1)
After the knee injury, he needs to wear a *brace* when running.	3.96	0.64
There are plenty of clothes in the large *basket* donated to the charity shop.	5	0.25
Sarah is very meticulous and pays attention to every *detail* of her work.	2.5	0.96
Brokers need to detect any change in the *mood* of their clients.	1.75	0.14

#### Single-word variables

For each target word, we also retrieved multiple single-word semantic characteristics: for the concreteness effect, we retrieved the ratings from Brysbaert et al. (2014), as well as a measure of composite sensorimotor strength from Lynott et al. (2020) as a possible alternative. Following observations made by Pollock (2018), we retrieved also valence and arousal (Warriner et al., 2013), as the emotional valence and arousal might differ between concrete and abstract words and could therefore act as confounds. We retrieved also a number of possible lexical psycholinguistic covariates: OLD20 (average Levenshtein distance computed over all word forms in the English CELEX lexical database), reaction times and accuracy in lexical decision task from the British Lexicon Project (Keuleers et al., 2012); orthographic neighbourhood size (Coltheart’s N), unconstrained bigram and trigram frequency from the CELEX database retrieved from McWord (Medler & Binder, 2005, http://www.neuro.mcw.edu/mcword/); frequency and log-frequency (Zipf) from the SUBTLEX-UK database (van Heuven et al., 2014). We included variables about the more general sentence context, such as the position of the word in the sentence; frequency, log-frequency (Zipf) and the number of letters of the preceding word from the SUBTLEX-UK database; word2vec average similarity between the target word and the preceding content words (Mikolov, Chen, et al., 2013). As exploratory measures, we looked at semantic diversity (Hoffman, Lambon Ralph, et al., 2013) and age of acquisition (Kuperman et al., 2012) as possible alternatives for a word’s concreteness. As this was decided post-hoc, we could retrieve the semantic diversity for only 397 out of 400 target words.

In Figure 1 we report the intercorrelation matrix for the variables that were considered, and in Table 2 a summary of their characteristics. Measures refer to the target word, unless indicated as *PRECEDING* indicating that it refers to the word before the target. To avoid collinearity among the predictors, we included only a subset of the currently presented predictors in the analysis, based on their estimated coefficients in the models.

**Table 2.** Summary statistics of psycholinguistics and semantic variables. In bold are highlighted the variables included in our analyses.

	**Concreteness**	**Length**	Bigram Frequency	Trigram Frequency	Orthographic Neighbour-hood Size	OLD20	Frequency	**Zipf Value**	**Valence**	**Arousal**	**Sensorimotor strength**
mean	3.25	5.51	21460.18	2344.83	3.34	1.86	5816.58	4.16	5.22	4.16	4.63
std	1.15	1.07	10782.10	2814.40	4.14	0.42	7390.54	0.55	1.35	1.00	0.93
min	1.19	4.00	3221.93	18.34	0.00	1.00	81.00	2.61	1.68	2.15	1.92
max	5.00	7.00	68055.67	28695.40	19.00	3.45	51028.00	5.40	8.05	7.05	7.22
	**Age of Acquisition**	**Semantic Diversity**	Context Similarity	PRECEDING Frequency	PRECEDING Zipf Value	PRECEDING Length	**Position**	Cloze Probability	**Plausibility**	**Predictability**	
mean	7.86	1.70	0.15	2505782.39	5.92	4.64	8.46	0.28	5.78	0.43	
std	2.32	0.25	0.08	3634761.17	1.42	2.73	1.55	0.32	0.70	0.28	
min	2.5	0.74	0.01	11.00	1.77	1.00	6.00	0.00	2.80	0.02	
max	15.56	2.29	0.55	9418422.00	7.67	13.00	12.00	1.00	6.77	1.00	

The pattern of correlations among the various predictors was generally as expected. More specifically, concreteness was correlated with sensorimotor strength, semantic diversity, and age of acquisition (concrete nouns are associated with stronger sensorimotor experiences; abstract words are more diverse and learnt later in time). We consider concreteness as the primary focus of this study; but, as sensorimotor strength is related and has already been shown to affect reading times (Juhasz, 2018), we explored also its effect. The number of characters in a word is correlated with two measures of orthographic similarity, Coltheart’s N and OLD20, which are in turn related to each other. Bigram and Trigram frequency are correlated because they measure similar orthographic features. Frequency and LogFrequency (Zipf) are strongly correlated to each other. They also are correlated with the RTs and accuracy in lexical decision as measured in the British Lexicon Project. Finally, predictors about context are correlated with each other, including context semantic similarity (with the target word), cloze probability, and semantic predictability. The length and frequency measures describing the word immediately preceding the target word are strongly correlated with each other.

### Procedure

Stimuli were created and presented using the PsychoPy (Peirce et al., 2019) and PyGaze (Dalmaijer et al., 2014) toolboxes. All stimuli were presented on a uniform grey background, on a single line in 20-px monospaced font (Consolas) in black colour. Each sentence was aligned to the centre of the screen. The monitor had a resolution of 1280 × 1024px and a refresh rate of 60 Hz. Participants were seated approximately 70 cm away from the screen. Vision was binocular, but eye movements were recorded from the right eye only, using an EyeLink 1000 Plus eye tracker (SR Research, Toronto, ON, Canada) with a sampling rate of 1000 Hz. A 9-point calibration procedure was performed before the start of each block and when necessary within blocks. Before the start of the study, participants were familiarised with the calibration procedure and with the task with four practice sentences and one question. The experiment lasted no longer than 1.5 h.

Each trial started with a fixation cross on the left side of the screen. Participants had to fixate on that cross to trigger the appearance of a sentence. Once they finished reading the sentence, they were instructed to look at a black square on the right bottom corner of the screen, to finish each trial. After 10% of trials, some simple yes/no comprehension questions were presented to check whether participants were attentive and that they were reading for comprehension. Participants were asked to press one of two keys to make their response. All participants completed the task successfully, as the comprehension questions were responded accurately on average 95.2 ± 3.9% (range 85-100). The experiment was divided into 10 blocks, each one containing 40 sentences; the order of presentation of the sentences was fully randomised.

### Data analysis

The study was pre-registered on the Open Science Framework repository: https://osf.io/qzksv.

Data were pre-processed in Python (version 3.6), while the statistical analyses were conducted in R (version 3.6.2). Scripts for the pre-processing and analysis are available in the repository https://github.com/magna-fede/EOS We relied on EyeLink’s algorithm for event detection, and we focused our analysis on first-pass fixation durations on each target word. Trials were not included in the analysis if either there was a track loss when fixating on the target word or the fixation of interest was immediately preceded or followed by a blink. We included in the analysis only fixations that lasted between 80 and 600 ms, excluding anomalous fixation durations from further analysis.

We adopted a multiple regression design with continuous variables and included possible confounds as covariates to ensure that our stimuli were as naturalistic as possible. Dichotomising our stimuli (i.e. a factorial design with factors concreteness and predictability with two levels each) might introduce other confounds (e.g. highly abstract words tend to be less frequent, less familiar, etc.). Furthermore, matching stimuli for a wide number of categories would have resulted in a smaller number of sentences. We tried to overcome these issues with a multiple regression design with participants and items as crossed random effects (Baayen, 2004; Baayen et al., 2008).

First-pass measures of interest were *first-fixation duration* (FFD), i.e. the duration of the first eye fixation on a target word, when it is fixated during first pass; *gaze duration* (GD), which is the sum of all the consecutive fixations made on the target word during first pass (i.e. not crossing word’s boundary); and *probability of fixation* (PrF), which reflects the likelihood of fixating a word, as opposed to skipping it during first pass. For each variable we fitted a linear mixed effect model, or a logistic mixed model for PrF, using the lme4 and lmerTest packages (Bates et al., 2015; Kuznetsova et al., 2017) with participants and items as crossed random effects. All independent variables were standardised. For each model, we checked which covariates influenced the dependent variable, and when they were significant predictors, they were included in the models. We investigated the contribution of predictability and concreteness, as well as their interaction. The random effects structure consisted of random intercept and slopes over participants for each covariate (assuming uncorrelated parameters for model convergence) and random intercept over items. Where appropriate, we removed terms with zero variance over the random slope (Barr et al., 2013). Packages from the easystats environment were also used for analyses and visualisation (Makowski et al., 2020).

### Exploratory analyses

We ran additional exploratory analyses to test if any other context and meaning-level characteristics affected fixation durations (we tested the influence of valence, arousal, and plausibility). Moreover, we analysed other single-word characteristics reported in previous literature, namely semantic diversity (Hoffman, Lambon Ralph, et al., 2013), and age of acquisition (Kuperman et al., 2012).

## Results

A summary of the FFD, GD and PrF measures is reported in Table 3.

**Table 3.** Summary of the measures, averaged across participants.

	FFD (ms)	GD (ms)	PrF (%)
Mean (SD)	220.97 (24.60)	235.81 (31.38)	68.08 (14.56)

As a first step, we inspected which covariates significantly predicted each measure of interest (i.e. dependent variables were FFD, GD, or PrF; on this step, no context- and semantic-level variable was considered). The covariates included in each model are word frequency, ordinal position of the word in the sentence, and number of letters of the target word (length). Once we fitted the basic model for each measure, we inspected the contribution of our predictors of interest (i.e. predictability, concreteness, and their interaction). For all analyses, the frequency of the target word is included in the form of Zipf value.

The results for FFD, GD and PrF are reported in Table 4, Table 5, and Table 6 respectively.

**Table 4.** Results of the LME for the first fixation duration analysis.

Predictors	Estimates	CI	*p*
Intercept	220.7	212.98 – 228.41	**<0**.**001**
Frequency (Zipf)	-1.81	-3.93 – 0.30	0.093
Position	2.04	0.17 – 3.91	**0**.**033**
Length	0.32	-1.45 – 2.09	0.723
Predictability	-5.26	-7.18 − -3.33	**<0**.**001**
Concreteness	-2.07	-4.03 − -0.11	**0**.**038**
Predictability*Concreteness	-0.71	-2.47 – 1.05	0.429
Random Effects			
*σ*^2^	4198.86		
*τ*_00__ID_	145.51		
*τ*_00__Subject.4_	587.96		
*τ*_11__Subject.ConcM_	6.8		
*τ*_11__Subject.Sim_	5.17		
*τ*_11__Subject.Position_	5.53		
*τ*_11__Subject.LogFreqZipf_	15.18		
*ρ*_01_			
*ρ*_01_			
ICC	0.03		
*N*_Subject_	40		
*N*_ID_	400		
Observations	10966		
Marginal *R*^2^ / Conditional *R*^2^	0.011 / 0.046		

Significant predictors (α=0.05) have been highlighted with *p*-value in bold.

**Table 5.** Results of the LME for the gaze duration analysis.

Predictors	Estimates	CI	*p*
Intercept	234.99	225.10–244.89	**<0**.**001**
Frequency (Zipf)	-3.1	-5.98 − -0.21	**0**.**035**
Length	4.94	1.90 – 7.99	**0**.**001**
Position	2.42	-0.13 – 4.97	0.062
Predictability	-8.64	-11.50 − -5.78	**<0**.**001**
Concreteness	-1.22	-4.06 – 1.63	0.402
Predictability*Concreteness	-0.64	-3.14 – 1.86	0.615
Random Effects			
σ^2^	7103.8		
*τ*_00__ID_	346.53		
*τ*_00__Subject.5_	955.29		
*τ*_11__Subject.ConcM_	17.14		
*τ*_11__Subject.Sim_	17.15		
*τ*_11__Subject.Position_	4.89		
*τ*_11__Subject.LEN_	29.93		
*τ*_11__Subject.LogFreqZipf_	22.64		
*ρ*_01_			
*ρ*_01_			
ICC	0.05		
*N*_Subject_	40		
*N*_ID_	400		
Observations	10966		
Marginal *R*^2^ / Conditional *R*^2^	0.018 / 0.066		

Significant predictors (α=0.05) have been highlighted with *p*-value in bold.

**Table 6.** Results for the probability of fixation logistic regression analysis.

Predictors	Odds Ratios	CI	*p*
Intercept	2.71	2.12 – 3.48	**<0**.**001**
Frequency (Zipf)	0.94	0.90 – 0.99	**0**.**017**
Length	1.42	1.34 – 1.50	**<0**.**001**
Position	0.96	0.92 – 1.00	0.069
Predictability	0.92	0.88 – 0.96	**<0**.**001**
Concreteness	0.98	0.93 – 1.03	0.473
Predictability*Concreteness	1	0.95 – 1.04	0.839
Random Effects			
*σ*^2^	3.29		
*τ*_00__ID_	0.04		
*τ*_00__Subject.3_	0.62		
*τ*_11__Subject.ConcM_	0.01		
*τ*_11__Subject.LEN_	0.01		
τ_11__Subject.LogFreqZipf_	0		
*ρ*_01_			
*ρ*_01_			
ICC	0.01		
*N*_Subject_	40		
*N*_ID_	400		
Observations	15676		
Marginal *R*^2^ / Conditional *R*^2^	0.041 / 0.055		

Significant predictors (α=0.05) have been highlighted with *p*-value in bold.

**Figure 2.**
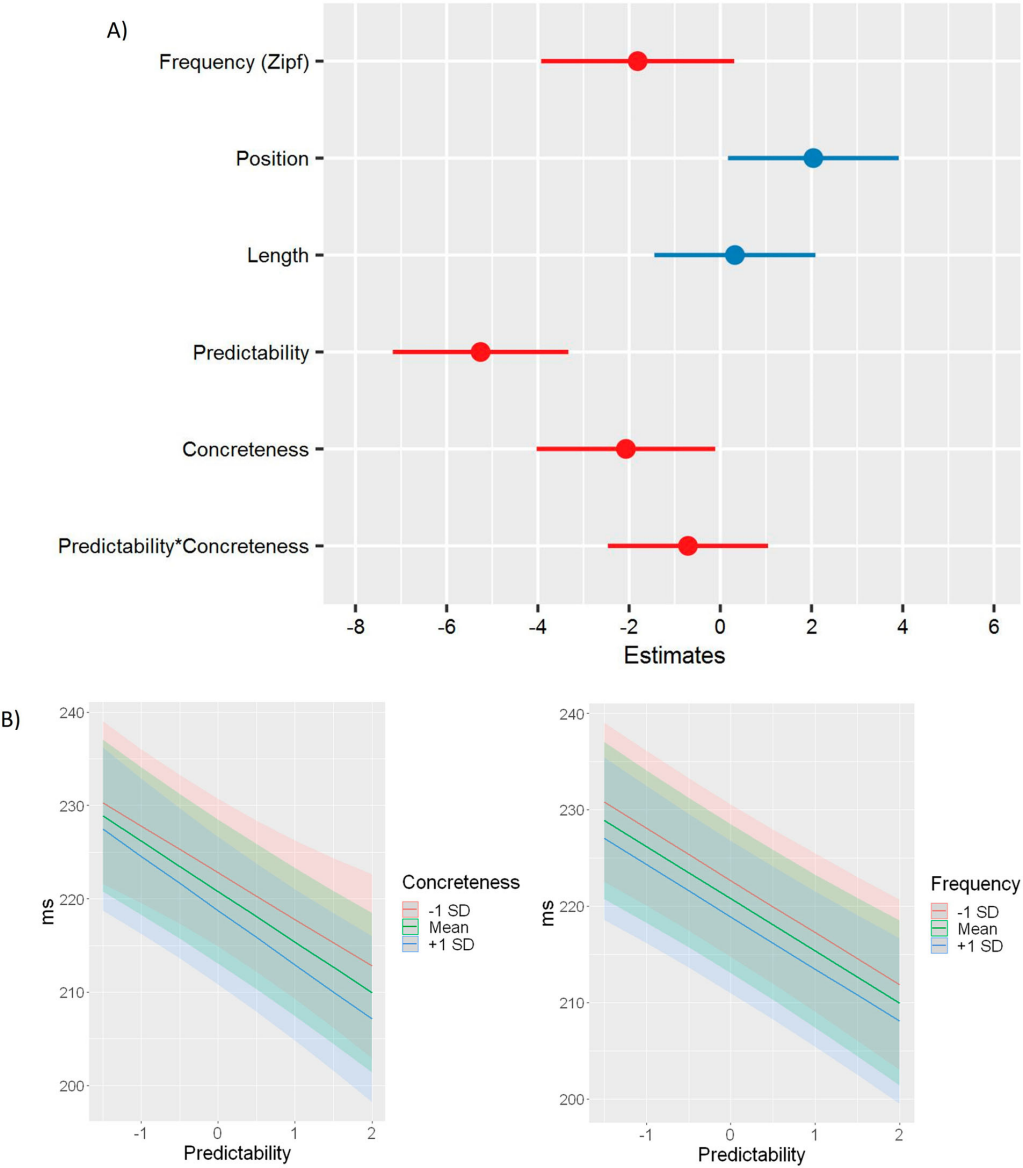
(A) Visual representation of the estimates in the model. (B) Comparison between conditional effects of concreteness and frequency in the full FFD model, when keeping the other predictors constant.

In the FFD analysis, we removed the random slope for the interaction Predictability:Concreteness to avoid singularity, as it had variance = 0 (Barr et al., 2013). FFD was affected by both concreteness (*p* < 0.05) and predictability (*p* < 0.001). However, there was no evidence of an interaction between the two parameters. As expected, unpredictable words received longer fixations than predictable words and abstract words were fixated longer than concrete words. Importantly, the magnitude of the predictability effect was much larger than that of concreteness. The frequency effect was marginally significant (*p* < 0.1) and we observed an effect of ordinal position (*p* < 0.05). Length did not predict FFD. All the predictors, except position, had a facilitatory effect – larger values were associated with shorter FFDs (while for position, words that were preceded by a larger number of words were associated with longer FFDs) (Figure 2(A)). In order to better understand the relationship between predictability and concreteness as well as frequency, we plotted the predicted FFD values for the predictors of interest, while keeping constant all the other parameters in the model (Figure 2(B)). The plots support the additive effect between concreteness and predictability, but also qualitatively show that in our stimuli the effects of concreteness and frequency on FFD are of comparable magnitude.

In the GD analysis (Table 5), we also removed the random slope for the interaction Predictability:Concreteness as it had variance = 0. In this case, we did not observe any effect of concreteness, nor an interaction between concreteness and predictability. We replicated the frequency effect (*p* < 0.05) that relatively high-frequency words received shorter fixations than less frequent words. The position of the target word only marginally influenced GD (*p* < 0.1). Finally, contrary to FFD, the length of the target word was a significant predictor of gaze duration (*p* < 0.001), as longer words were associated with longer GDs.

Finally, we fitted a logistic regression model to the probability of fixating a word (Table 6). In this case, we replicated the finding that a word’s predictability (*p* < 0.001), frequency (*p* < 0.05) and length (*p* < 0.001) affect the probability of fixating a word. Concreteness was not a significant predictor. Position was a marginally significant predictor. Specifically, predictable, shorter, and more frequent words are more likely to be skipped relative to unpredictable, longer, less frequent words.

To sum up, we found additive effects of concreteness and predictability on FFD, but no evidence for interactive effects of the two variables. While the effect of predictability persisted in GD and PrF, the effect of concreteness (which was small in FFD) was not significant in those cases. Importantly, the additive relationship between concreteness and predictability parallels previous findings about frequency and predictability.

Considering that the effect of concreteness was rather small (and present only in one of the three measures under consideration), and after the suggestion of one anonymous reviewer, we decided to validate our results on the Provo Corpus (Luke & Christianson, 2018), an openly available eye-tracking dataset that contains word-by-word cloze ratings. Detailed information and discussion can be found in the Appendix. In sum, we found that when accounting for different word classes, the results are compatible with the results in our study.

### Exploratory analysis

We carried out additional analyses to (1) test how robust our findings are when considering other potentially confounding variables; (2) test whether there are other characteristics which could be explored in more depth in future studies. First, we examined the possible influence of context variables other than predictability; then, we investigated whether other single-word semantic variables influenced FFD. The exploratory analyses reported below were conducted on FFD measures, as we found a reliable additive influence of concreteness and predictability only for FFDs.

Results of the analysis on whether any other context variable influenced FFDs are reported in Table 7. Interestingly, we found that sentence plausibility had an effect on FFD that was independent of predictability, despite being highly correlated (0.57); while this has been observed in previous EEG literature (Nieuwland et al., 2020), it has not been reported in eye-tracking studies before. Importantly, the inclusion of plausibility did not undermine the role of concreteness as a predictor but indeed corroborated its influence on FFD.

**Table 7.** LME model including all predictors that significantly influenced FFD.

Predictors	Estimates	CI	*p*
Intercept	220.65	212.93 – 228.37	**<0**.**001**
Frequency (Zipf)	-1.71	-3.82 – 0.40	0.113
Position	2.03	0.16 – 3.89	**0**.**033**
Lenght	0.37	-1.39 – 2.13	0.678
Predictability	-3.84	-6.04 − -1.65	**0**.**001**
Concreteness	-2.61	-4.60 − -0.61	**0**.**010**
Plausibility	-2.51	-4.90 − -0.11	**0**.**040**
Predictability*Concreteness	-0.47	-2.23 – 1.29	0.600
Random Effects			
σ^2^	4191.65		
*τ*_00__ID_	142.39		
*τ*_00__Subject.5_	588.71		
*τ*_11__Subject.plausibility_	11.68		
*τ*_11__Subject.ConcM_	6.46		
*τ*_11__Subject.Sim_	1.23		
*τ*_11__Subject.Position_	5.54		
*τ*_11__Subject.LogFreqZipf_	15.11		
*ρ*_01_			
*ρ*_01_			
ICC	0.04		
*N*_Subject_	40		
*N*_ID_	400		
Observations	10966		
Marginal *R*^2^ / Conditional *R*^2^	0.012 / 0.047		

Significant predictors (α=0.05) have been highlighted with *p*-value in bold.

Next, we explored if other single-word semantic variables affected FFDs. We separately included arousal and valence in the full model (i.e. the one presented in Table 4), to account for possible emotional confounds. However, neither of them influenced FFD.

We then tested possible alternative candidates for the concreteness effect. We started by examining the sensorimotor strength effect, as this variable is strongly related to concreteness and previous studies found that it influences FFD in compound words (Juhasz, 2018). We did not replicate this finding. Then, we performed the same analysis on two variables that tend to be highly correlated with concreteness: semantic diversity (Hoffman, Lambon Ralph, et al., 2013) and age of acquisition (Kuperman et al., 2012). While the effect of age of acquisition was marginally significant, the effect of semantic diversity was not. Finally, we also tested the influence of our participants’ age (considering that we had a wide range among participants), but it did not significantly influence FFDs.

## Discussion

We investigated whether single-word meaning (concreteness) and sentence context (predictability) interactively affected fixation durations during natural reading. Our sentences were constructed to resemble naturalistic and plausible stimuli. Firstly, we corroborated findings about concreteness effects on first fixation durations (FFD) – albeit small – when controlling for other relevant psycholinguistic variables (Juhasz & Rayner, 2003). However, we found no evidence of such influence of concreteness on gaze durations (GD) or skipping rates. These results were supported by an additional analysis performed on the Provo Corpus that produced compatible results (see Appendix for details). Finally, we did not find evidence of an interaction between concreteness and predictability on any of the measures analysed. Interestingly, this absence of an interaction parallels previous findings about the additive influence of word frequency and predictability on eye movements.

### Single-word semantic influences on FFD

We tested the robustness of the concreteness effect on FFDs for several possible confounding variables, beyond the lower-level covariates. For example, emotional features such as valence and arousal have been reported to affect single-word semantic features (Kousta et al., 2011). However, they did not affect the general pattern of our results.

We also investigated the nature of the concreteness effect by including variables that are correlated with concreteness, such as sensorimotor strength, semantic diversity, and age of acquisition. However, we found that they were worse predictors of FFDs than concreteness. Thus, concreteness was a better predictor of FFD than either sensorimotor strength, semantic diversity, or the age of acquisition. The specific reason why abstract words are more difficult to process than concrete words is still debated, but it seems that concreteness ratings (Brysbaert et al., 2014) capture some cognitive process that no other of the obvious alternative psycholinguistic variables is able to capture.

While being statistically reliable, the effect of concreteness, when controlling for predictability, was quite subtle in our analysis. However, we observed similar results when analysing the larger Provo Corpus dataset, where concreteness was a marginally significant predictor of FFD. It is important to highlight that while our study was designed for testing single-word semantic influences, the Provo Corpus was designed for other purposes and it is hard to fully control for all relevant confounds present in that dataset (see Appendix for more detailed discussion). Also, previous literature on semantic effects on eye movements is inconsistent, and here we report results following a pre-registered analysis protocol.

There seems to be a discrepancy between the eye movement literature and studies using conventional two-alternative forced-choice tasks. For example, in lexical decision tasks, larger concreteness effects have consistently been reported (e.g. Levy-Drori & Henik, 2006; Schwanenflugel et al., 1988; Van Hell & de Groot, 1998; but see Barber et al., 2013; Kousta et al., 2011 for a reversed concreteness effect). The cognitive processes that underlie concreteness may be sensitive to task demands. For example, Farahibozorg et al. (2022) reported a reaction time advantage for concrete words of about 100 ms in a semantic decision task, while in a lexical decision task, this difference was only 6 ms. Natural reading is arguably closer to semantic decision since we normally read to extract meaning from text. The fact that we found a relatively small concreteness effect in our eye-tracking data is therefore surprising. Further exploration and combination of methods might reveal more information about the neural and cognitive substrates underlying concrete and abstract words.

It is still an open question why the effect was present only in First Fixation Durations and not in Gaze Duration (both in our dataset and in the Provo Corpus, although in the latter the effect was just marginally significant). Concreteness may only affect the earliest stages of word processing and therefore have the largest effect on FFDs, while effects on GDs may be dominated by other effects. Previous EEG and MEG studies have shown that concreteness affects brain responses at early and late latencies (Farahibozorg et al., 2022), but it is not clear yet which of these effects influence behavioural responses such as eye movements. Future work may address these issues with a factorial design to maximise the likelihood of detecting a concreteness effect. Although investigating the possible interaction between predictability and concreteness was one of this study’s goals, we could not have predicted the relative size of predictability and concreteness effects with respect to each other in our dataset. We found a much stronger effect of predictability relative to the effect of concreteness, making it hard to detect any interaction. Future studies might consider contrasting different levels of predictability (e.g. excluding extreme values of predictability) to prevent this confound.

Also, frequency only had a marginal effect on FFDs. We argue that this reflects experimental design choices, as our paradigm was primarily designed to investigate the effects of concreteness and predictability. Thus, we had very few extreme frequency values, especially not very high-frequency words to avoid confounds with concreteness, as high-frequency words are more often concrete rather than abstract. Nevertheless, the direction of our frequency effect is consistent with previous findings and it was significant in GDs.

Overall, our findings about concreteness effects on early fixation durations are aligned with previous studies, showing that a word’s meaning affects early reading times as measured by eye movements in a natural reading paradigm. However, the results still need to be corroborated and extended in future studies, especially regarding the relationship between concreteness and predictability. Ultimately, future research should establish a link between these effects on eye movements and the underlying brain processes.

### Relationship between context and single-word semantics

We did not find evidence in support of an interaction between concreteness and predictability on any of the measures of interest. This is in apparent contrast with previous early reports of a mitigation in the difficulty associated with abstract words following a predictive context (Schwanenflugel et al., 1988; Schwanenflugel & Shoben, 1983; Schwanenflugel & Stowe, 1989). It is possible that the differences between the task employed in the two experimental situations were too large (i.e. natural reading vs. naming/lexical decision), or that the interactive effect depends on a time delay between the context and the target word (which is smaller in natural reading). Another plausible alternative is the difference between the stimuli used: in our task, we used naturalistic sentences, while some of the above-mentioned studies did not. For example, in the naming experiment reported by Schwanenflugel and Stowe (1989), an exemplar of the unpredictable sentences’ structure was “You'll never guess that the last word of this sentence is // values.” (where the last word was presented separately). Arguably, the content of such a sentence differs from that of a typical sentence in naturalistic settings and also differed from the predictable sentences condition in the same experiment. Even though the interaction terms approached significance in the Provo Corpus analysis, this should be interpreted with care due to the disproportionate representation unpredictable relative to predictable sentences (making concreteness predicted values unreliable in those cases) and due to imbalanced representation of abstract/concrete words across different word classes.

An additive influence of concreteness and predictability on FFDs would parallel the additive influence found for word frequency and predictability. In Staub (2015) some alternative explanations for the additive, rather than interactive, effects of word frequency are proposed: Predictability and frequency may operate at different processing stages, with predictability effects starting when the target word is in parafoveal location, while frequency may affect processing at later stages (Staub & Goddard, 2019). Alternatively, word frequency and predictability effects may relate to different processes: while the former might influence lexical access because of differences in memory strength for low- vs. high-frequency words, the latter is proposed to change the probability of encountering some words over others. In principle, analogous interpretations can also account for the absence of an interaction between concreteness and predictability: concreteness and predictability might be influencing word processing at different stages or for different cognitive computations. However, an interaction of two variables can still occur when the corresponding information is processed at different stages and in different brain systems, as long as both variables affect the response and the effect of one variable depends on the size of the effect of the other variable (Norris et al., 2000). Thus, future studies should address this issue using a different methodology.

Behavioural measures derived from eye tracking, such as fixation duration, only inform us about the endpoint of a combination of processing stages until the moment of a behavioural response (i.e. saccade to another word is made). In contrast, EEG and MEG enable us to distinguish between different processing stages with millisecond precision. For example, previous research using serial visual presentation (i.e. not natural reading) showed that the N400 is modulated by concreteness (e.g. Barber et al., 2013; Holcomb et al., 1999) during sentence comprehension. Notably, in Holcomb et al. (1999), the authors observed an interactive effect between sentence context and concreteness (in the N400 time window), so that the concreteness effect was present in anomalous sentences, but not in congruent sentences. This might indicate that context support might reduce the difference in processing abstract and concrete words. This would also be consistent with previous interactive effects found in ERP studies manipulating frequency and predictability/context support (Dambacher et al., 2006; Van Petten & Kutas, 1990). Indeed, the absence of an interaction in behavioural paradigms does not rule out a possible functional interaction, which only neural measures can detect. Future studies should aim at exploring these issues with naturalistic reading co-registration paradigms (Degno & Liversedge, 2020; Dimigen et al., 2011).

The co-registration of eye movements with EEG/MEG could enable us to study the brain processes that support natural reading while still maintaining a good amount of experimental control (Degno et al., 2021; Dimigen & Ehinger, 2021; Weiss et al., 2021). The combination of simultaneous behavioural responses (fixation durations) and brain activity (generated by each fixated word) using novel connectivity and multivariate pattern-based methods (Farahibozorg et al., 2022; Huth et al., 2016; Rahimi et al., 2022) could unravel the dynamics of the semantic brain networks during natural language processing.

## Supplementary Material

Supplemental Material
