# Supplementary material for: An eye on semantics: a study on the influence of concreteness and predictability on early fixation durations
Source: Lang Cogn Neurosci. 2023 Nov 9;39(3):302–16. doi: 10.1080/23273798.2023.2274558 (PMC10962710; doi:10.1080/23273798.2023.2274558)
Supplement: Supplemental Material [file PLCP_A_2274558_SM6234.docx]

# Appendix

Given that the concreteness effect we found was relatively small and present only in first fixation durations (FFD) (but not in gaze durations (GD) or the probability of fixating a word), we attempted to validate our results exploring the relationship between concreteness and predictability effects in the Provo Corpus (PC) (Luke & Christianson, 2018)^[[1]](#footnote-1)^. This corpus consists of eye-tracking data from 84 participants as they read 55 short passages that were extracted from various sources (including news articles, magazines and works of fiction), for a total of 2,689 words. For each word in the corpus, cloze probability scores are available. While the amount of data in this corpus is extremely valuable, there are some important differences when compared to our study (referred to as EOS from now on). First, this corpus consists of natural text, and the sentences in it were not created with a specific scientific question in mind. In contrast, our sentences, although naturalistic, were specifically created for testing the relationship between the influence of context and single-word meaning on fixation durations. Thus, the variability in the data is likely to be greater in the PC and possible confounds may have not been carefully accounted for. This is partly reflected in the distribution of some of the psycholinguistic variables, especially cloze probability and concreteness which we will discuss in more detail, and the inter-correlations among variables (Figure 1A and 1B). Secondly, in EOS we only looked at nouns that were presented towards the end of the sentence, and we ran a cloze task only on them. In contrast, the PC contains predictability ratings for all words that were presented. To make full use of the corpus’ value, we are going to examine concreteness and cloze effects on all the content words in the corpus (1,043 words, after excluding words that were not fixated on first-pass), including their word class as a covariate; more specifically, we analysed reading times from 333 nouns, 380 verbs, 182 adjectives, and 148 adverbs. We decided to include word class as a covariate because a) there might be differences in the way each category’s concreteness is perceived; the way a noun is perceived as concrete might differ from the way an adjective or a verb is; and b) the distributions of both frequency and concreteness varied consistently across word categories (see Figure 1A). Thirdly, concreteness was not included in the PC metadata. To overcome this issue, we included concreteness ratings as used in the main study (Brysbaert et al., 2014). We will include in the analysis only those words for which it was possible to retrieve a concreteness rating. Finally, in our main analysis, we did not use simple cloze probability, but we computed a semantic predictability score that was described in the methods section. We will compute the same measure also in this case in order to validate its usefulness over simple cloze probability.

In Figure 1 we compare the predictors’ distributions grouped with respect to their word class between PC and EOS. The main differences are:

- EOS does not contain extremely frequent or infrequent words, contrary to the PC which contains many high-frequency words, especially adjectives and verbs. This is a possible explanation for why in EOS the well-established frequency effect on FFDs was only marginally significant. Note that the low variability in frequency in EOS was motivated by the necessity to control for possible confounds with concreteness as highly abstract words are also infrequent.
- PC contains relatively more words that are unpredictable, as reflected in both the (semantic) predictability score and the cloze probability distribution. While in both datasets the distribution is skewed towards unpredictable words, this tendency is more prominent in the PC.
- The correlation among variables was kept very low in the EOS dataset (when possible), in order to minimise possible confounds (Figure 1C). As the PC contains natural text, this was not the case (Figure 1B).
- The concreteness distribution was very different for different word categories: while nouns were rated as concrete in the vast majority of cases, adjectives, adverbs, and verbs were relatively more abstract.


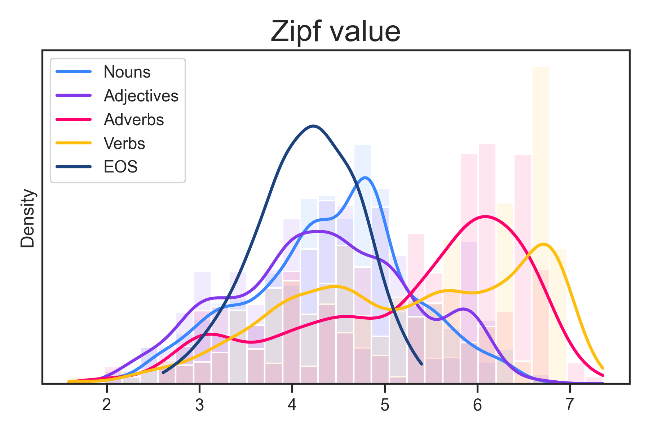

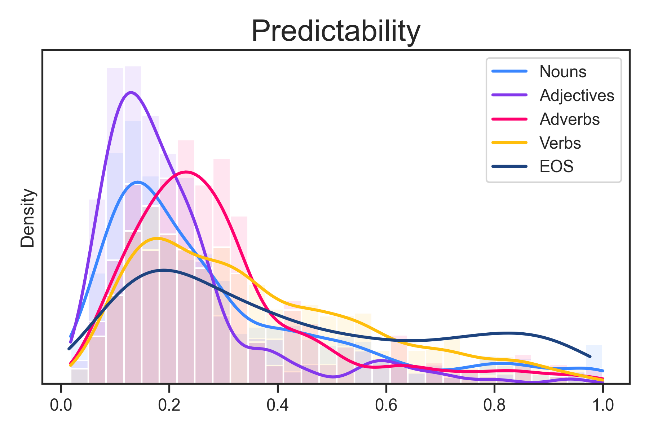

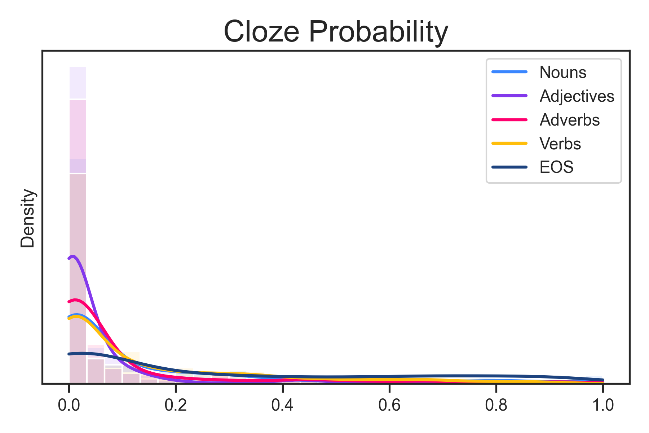

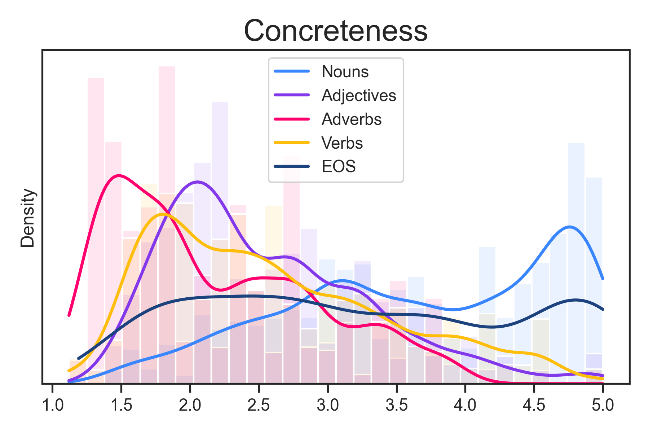


A


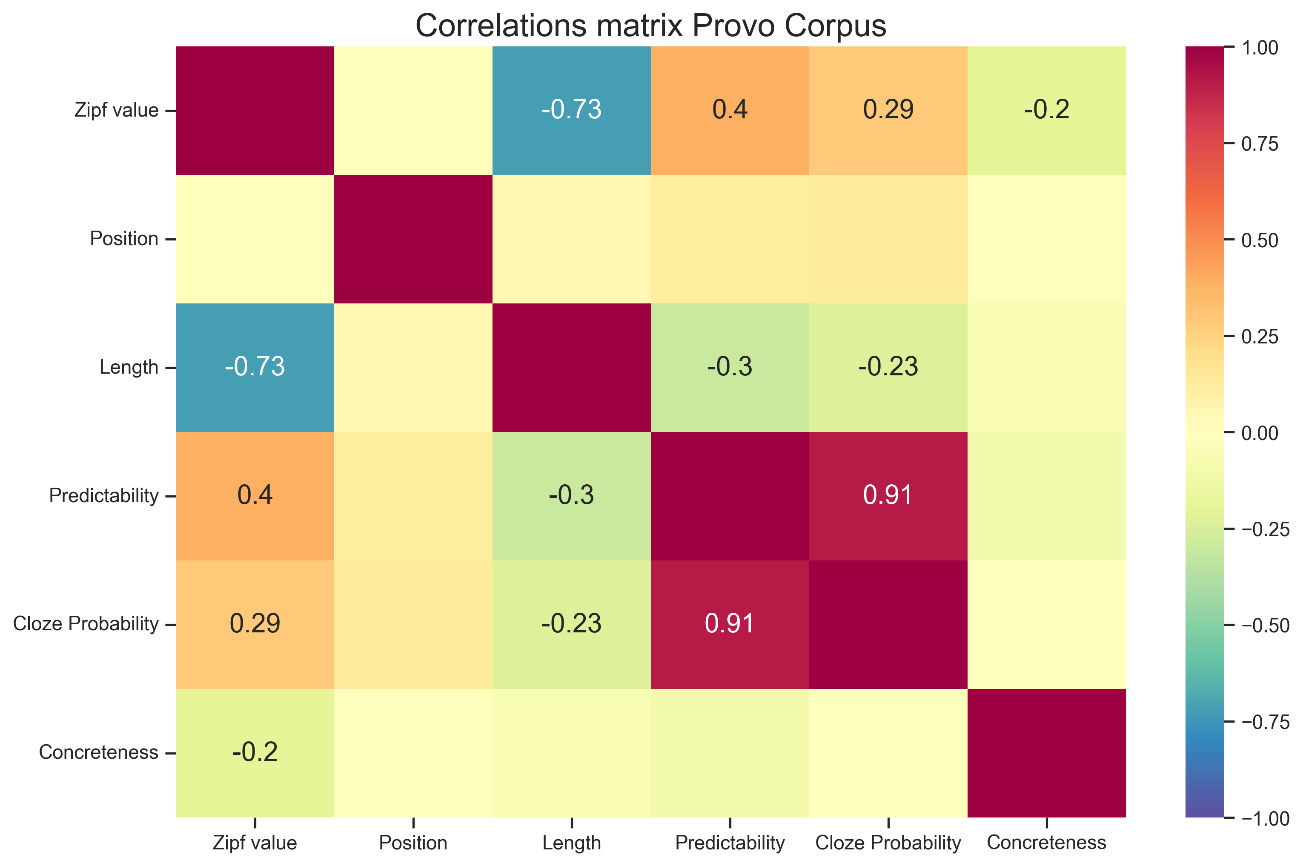


B

*
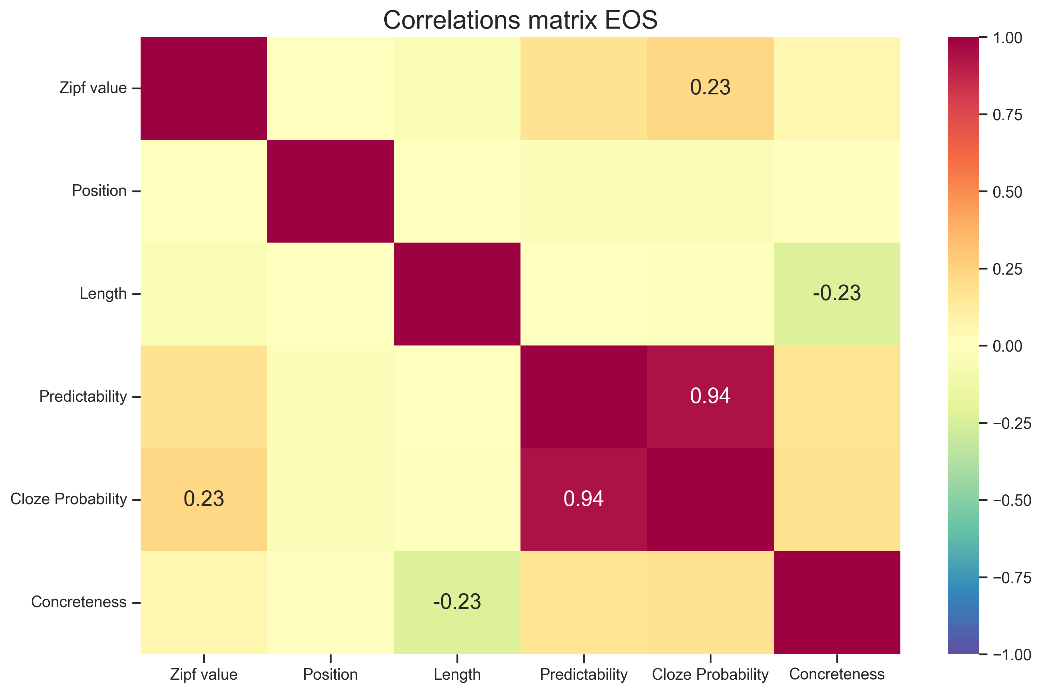
*

C

*Figure 1. A) Density distribution for investigated variables; the dark blue line represents the distribution in EOS dataset. B) Correlation among the psycholinguistic variables inspected in the PC analysis; reported numerically are correlations greater than* |*.2*|. C) *Equivalent of B for the EOS dataset; the full correlation matrix can be found in the main text in Figure 1*.

For the PC analysis, we largely followed similar preprocessing steps as in EOS, although some minor differences exist; for example, fixation duration inclusion criteria were relatively less stringent in PC (80-800 ms in PC as opposed to 80-600 in EOS). All predictors were normalised before fitting the model. Preprocessing of the data was performed using custom Python scripts, while statistical analysis was conducted in R (all code is available on the study repository <https://github.com/magna-fede/EOS/>).

We fitted the following models in order to test the validity of the concreteness effect:

- linear mixed effects (LME) model fitted on FFD with items and participants as crossed random effects with predictors Zipf value, word length, position in the sentence, and a three-way interaction between cloze probability score, concreteness, and word class;
- LME model fitted on GD with items and participants as crossed random effects with predictors Zipf value, word length, position in the sentence, and a three-way interaction between cloze probability score, concreteness, and word class.

We then fitted the same models but included (semantic) predictability rather than cloze probability as a predictor, so that models were specified as the models above but substituting cloze probability with semantic predictability. We did not include random slopes in the models as this resulted in singular fits or convergence issues.

The inspection of the parameters in the first model (Table 1A) showed that frequency, position in the sentence, and cloze score significantly influenced FFD. FFD was marginally predicted by concreteness, in a direction consistent with EOS. Word length was not a significant predictor. Interestingly, also the interaction between concreteness and cloze probability approached significance. While we will not interpret the specific interactions between concreteness and cloze probability for different word classes as this is beyond the scope of this analysis, this could motivate further studies into this potential difference: Concreteness, predictability and the interaction term differed at least partly across word categories. Finally, we performed a likelihood ratio test contrasting the model fitted with and without the concreteness term to further validate the role of concreteness. The test revealed that the model including the concreteness term should be preferred (p <0.05).

Finally, we tested the observed power with respect to the “Concreteness” predictor in both studies, to assess which one was better designed for finding an effect. The powerSim function of the simr package^[[2]](#footnote-2)^ (Green & MacLeod, 2016) showed that while the observed power of the predictor Concreteness is 39.00% in the PC analysis (34.70-43.43, 95% confidence interval), it reached 76% in the EOS study (72.01-79.68, 95% confidence interval).


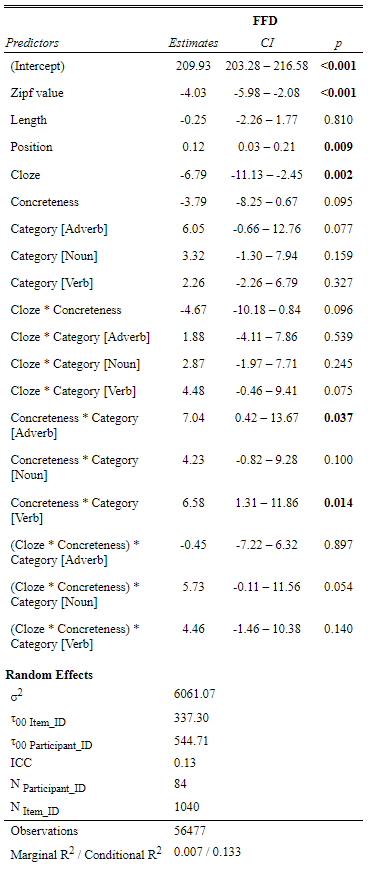

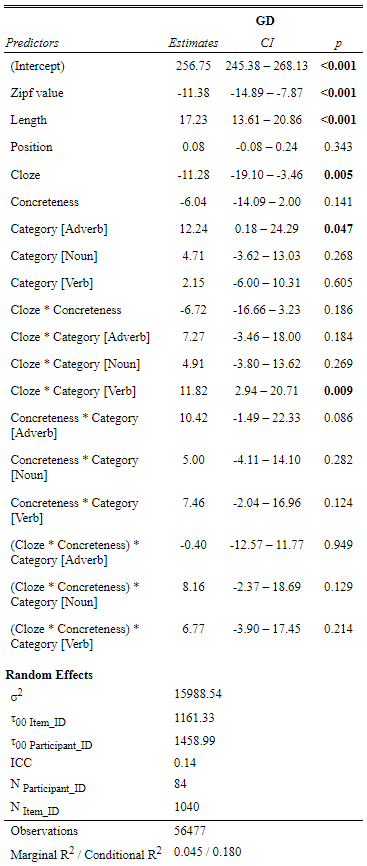


A

B

*Table 1 A) Results for the FFD analysis B) Results for the GD analysis.*

Figure 2A visually represents the relationship between concreteness and cloze probability for FFD and GD separately. In both cases, the high variability in the data should be highlighted. For both FFD and GD, it can be observed that the error increases significantly for highly predictable sentences; this is not surprising considering that there are fewer examples of that. The variability in estimated FFD is especially large in both adjectives and adverbs. Verbs lack examples of concrete words, while nouns lack examples of abstract words, in both cases resulting in very large variability on the respective estimated values. Figure 2B shows the relationship between frequency and predictability. Although also in this case the estimation of the effects is noisy for high cloze words, the data do not seem to support any interactive effect, consistent with the existent literature.


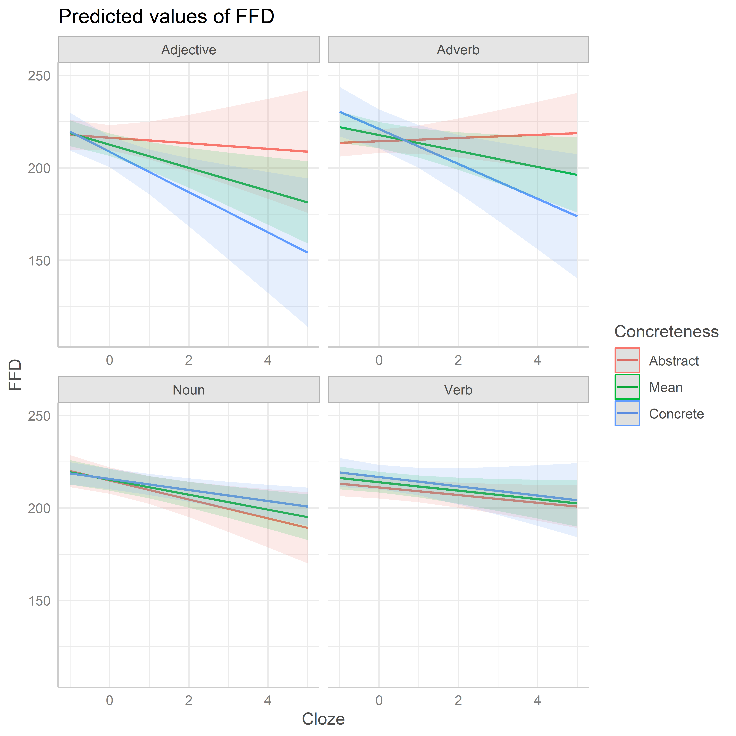

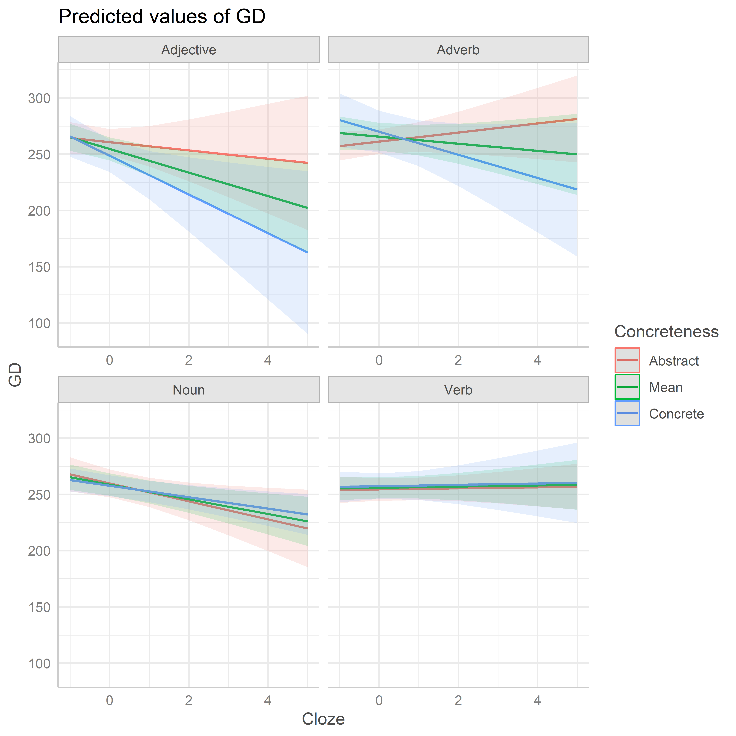


A


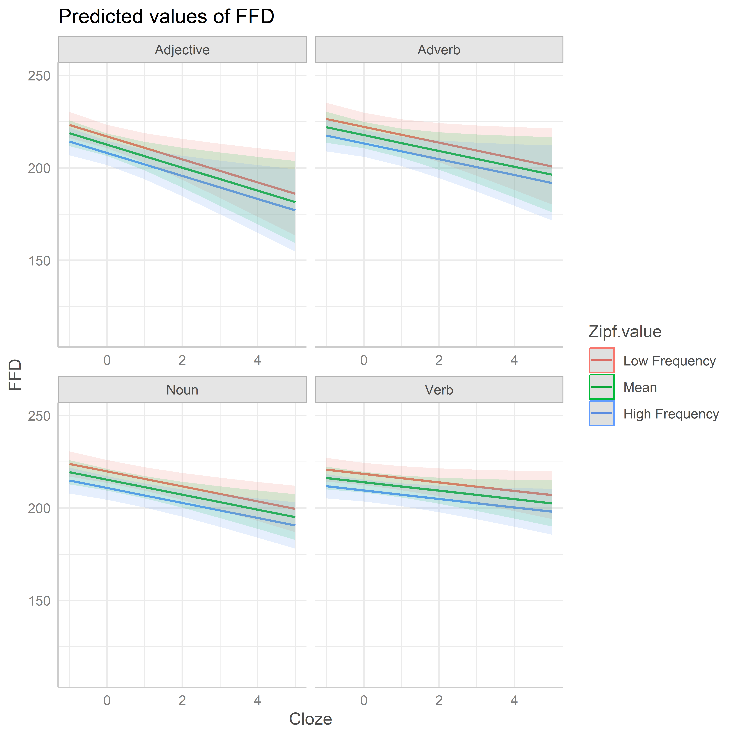

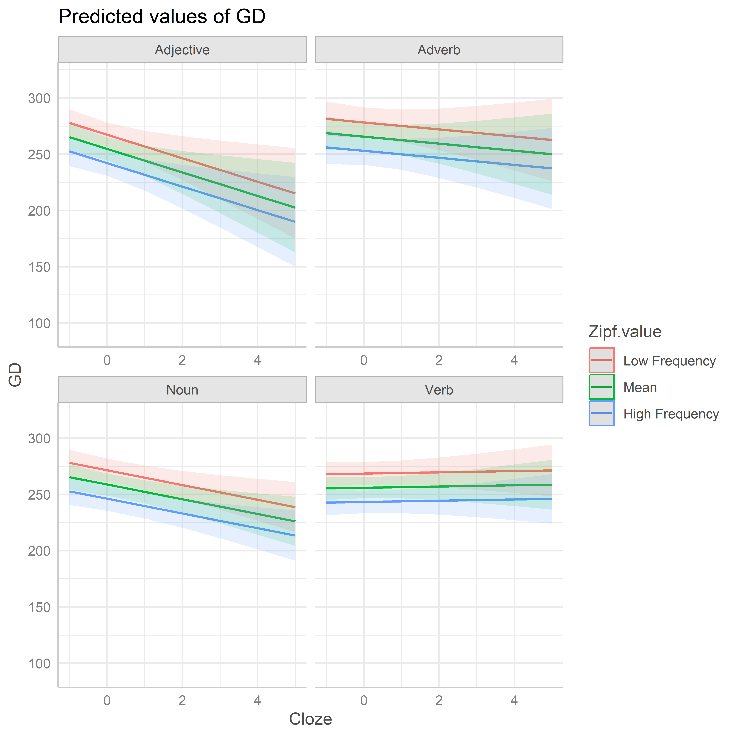


B

*Figure 2. The colour coding refers to +/- 1 standard deviation away from the mean of either concreteness or frequency. A) Concreteness * Predictability interaction across word classes. B) Frequency * Predictability interaction across word classes.*

Overall, the visual inspection of predicted values indicates that although the statistical test indicated a marginally significant interaction between concreteness and predictability (p<.1), it did not reach full significance due to the high variability in the data. The effect of concreteness seems to vary a lot across word classes, but we cannot be sure if this is due to the different distributions of the variables or a true interaction. Nevertheless, the effect of concreteness on FFD (and not on GD) although partially significant when examining the coefficients, it is significant in the model comparison test.


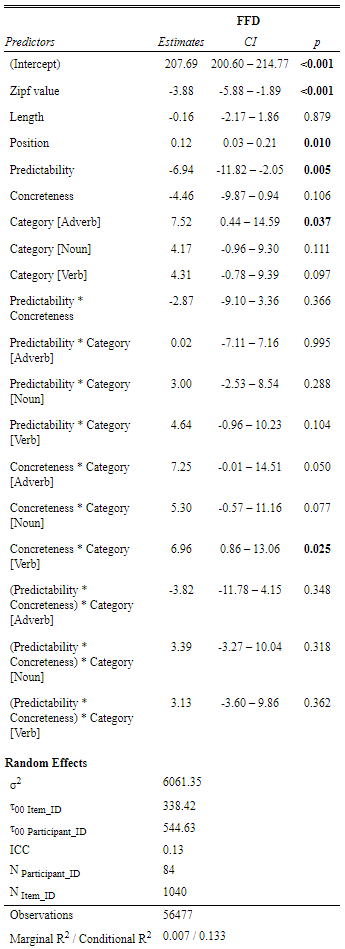

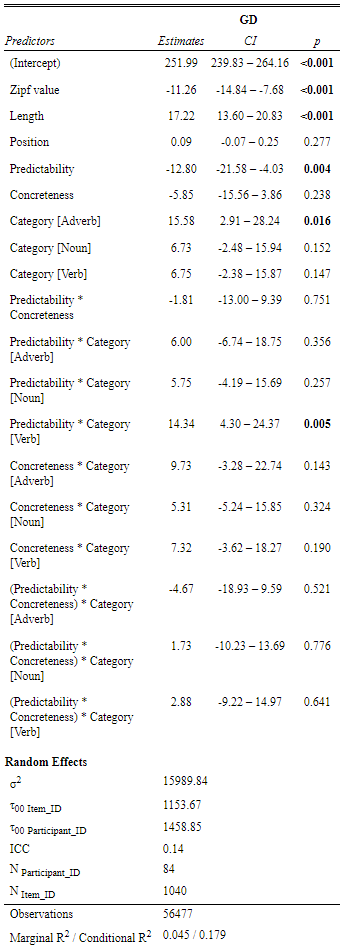


B

A

*Table 2 Models fitted using Semantic Predictability as a predictor instead of Cloze Probability A) Results for the FFD analysis B) Results for the GD analysis.*

This is consistent with what was found in the EOS study, and what was previously reported in the literature (Juhasz & Rayner, 2003).


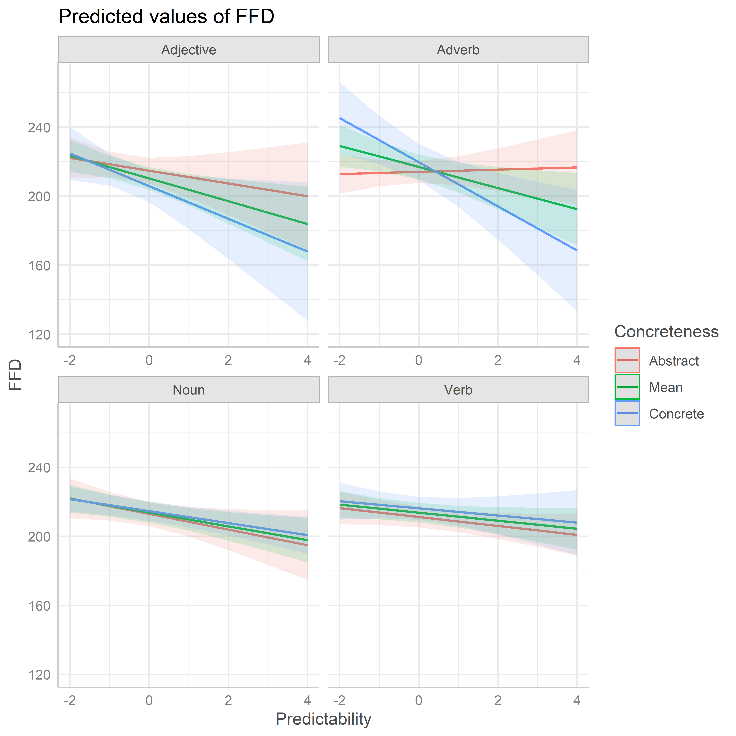

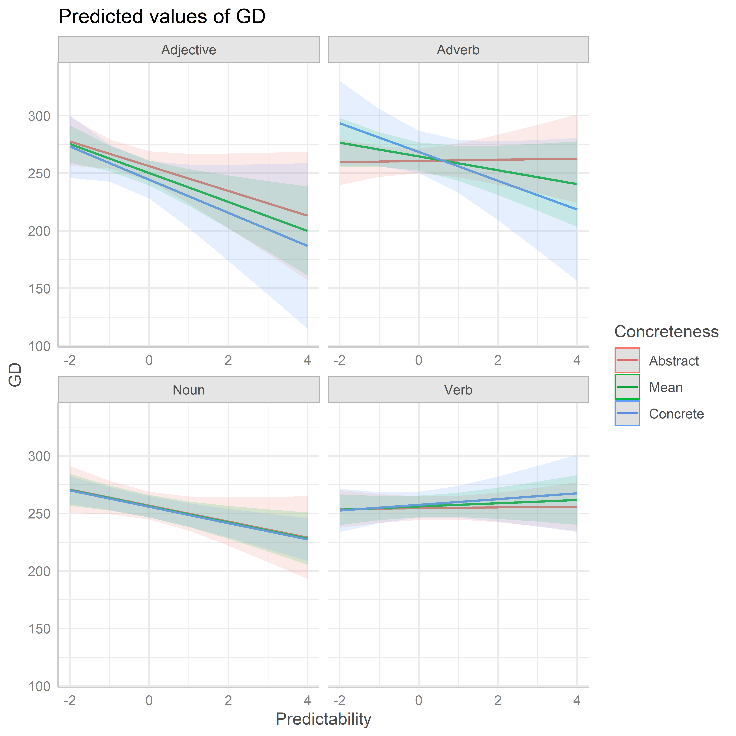

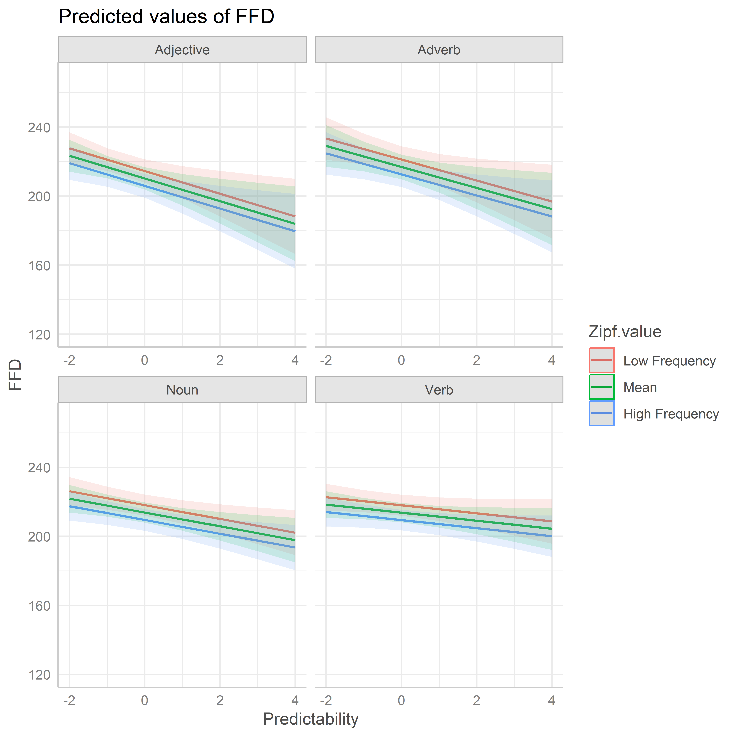

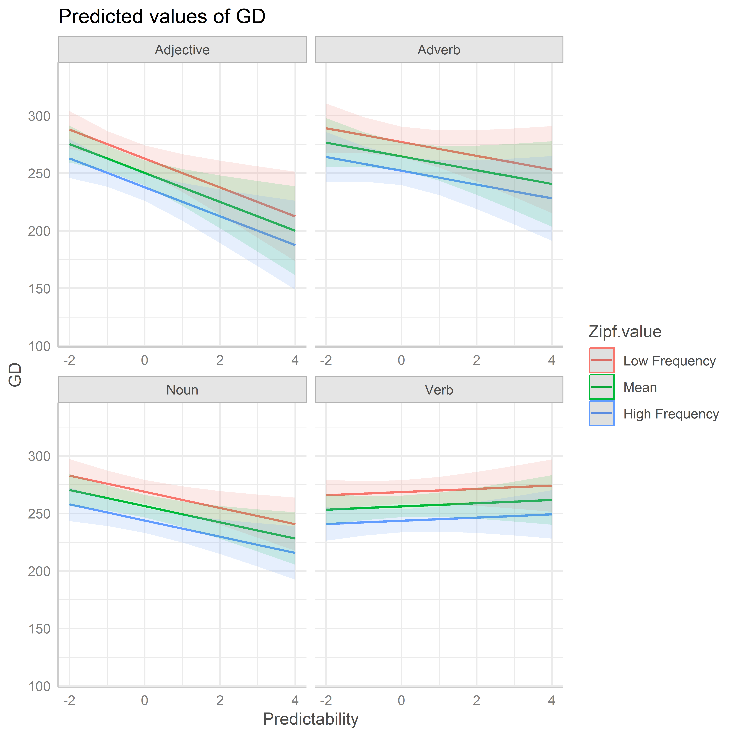


A

B

*Figure 3. The colour coding refers to +/- 1 standard deviation away from the mean of either concreteness or frequency. A) Concreteness * Predictability interaction across word classes in the model including Semantic Predictability. B) Frequency * Predictability interaction across word classes.*

We then substituted the cloze probability term with our semantic predictability score (Table 2A for FFD results and Table 2B for GD results). In practice, there was no benefit on either the variance explained by the model and it did not impact significantly on the other predictors, although it relatively reduced concreteness influence and the interaction term. Cloze probability and predictability were highly correlated (r=0.9). One of the initial motivations for computing this measure was to mitigate the skewness of the cloze probability score. While the benefit is clear in the predictor distribution plots for EOS, this is less impactful on PC (unpredictable words are still much more prominent than medium or highly predictable ones) (Figure 1A). Also, this adjustment does not result in reduced variability of the estimates (on the contrary the variability of the estimates seems greater) (Figure 3). The benefits of calculating semantic predictability as opposed to raw cloze probability, therefore, seem minimal or absent in the context of reading times.

Overall, the PC analysis results are compatible with what was found in the EOS: namely, FFD (but not GD) is affected by word position and marginally by concreteness. Word length affects GD but not FFD. Frequency and predictability affect both FFD and GD. The power analysis suggests that EOS’ design was more suited for finding an (albeit small) effect of concreteness (~76% observed power in EOS vs 39% in PC). Importantly, neither PC nor EOS seems adequate for testing the interaction between concreteness and predictability, although for different reasons. In PC this is likely due to a relatively high variability in the data caused by multiple factors, including the lack of either concrete or abstract words within each word class (when the data seem to support the proposal that word class has an impact on both the concreteness and the predictability effect). On the other hand, EOS power is affected by the disproportionate effect size of predictability compared to concreteness. Future studies interested in testing the influence of predictability on the semantic processing of single words can be informed by both EOS and PC findings and should A) test specific hypotheses using a factorial design rather than a regression design (at the potential expense of naturalness of the sentences); B) consider the relative magnitude of predictability effect when including highly predictable words (as in EOS), as this might confound an interaction with concreteness; and C) carefully consider which word class is the most suitable for the analysis.

1. We would like to thank an anonymous reviewer for this suggestion. [↑](#footnote-ref-1)
2. For PC, the observed power was calculated the model presented in Table 1A of the appendix, whereas for EOS, we used the model presented in Table 7 of the main text, excluding the interaction term which was not supported by the data. We used formula *powerSim(model, fixed("Concreteness”, "z"), nsim=500),* thus treating the observed t-values of the predictor concreteness as z-values, and running 500 simulations. [↑](#footnote-ref-2)
